# Production of both l‐ and d‐ *N*‐acyl‐homoserine lactones by *Burkholderia cepacia* and *Vibrio fischeri*

**DOI:** 10.1002/mbo3.1242

**Published:** 2021-11-15

**Authors:** Abiud E. Portillo, Elizabeth Readel, Daniel W. Armstrong

**Affiliations:** ^1^ Department of Chemistry and Biochemistry The University of Texas at Arlington Arlington Texas USA

**Keywords:** quorum sensing, enantiomeric metabolites, signaling molecules

## Abstract

Quorum sensing (QS) is a complex process in which molecules, such as l‐*N*‐acyl‐homoserine lactones (l‐AHLs), are produced as essential signaling molecules allowing bacteria to detect and respond to cell population density by gene regulation. Few studies have considered the natural production and role of the opposite enantiomers, d‐AHLs. In this work, production of d,l‐AHLs by *Burkholderia cepacia* and *Vibrio fischeri* was monitored over time, with significant amounts of d‐AHLs detected. Bioluminescence of *V*. *fischeri* was observed with maximum bioluminescence correlating with the maximum concentrations of both l‐ and d‐ octanoyl‐homoserine lactones (l‐ and d‐OHL). l‐Methionine, a precursor to l‐AHLs, was examined via supplementation studies conducted by growing three parallel cultures of *B*. *cepacia* in M9 minimal media with added l‐, d‐, or d,l‐methionine and observing their effect on the production of d,l‐AHL by *B. cepacia*. The results show that addition of any methionine (l‐, d‐, or d,l‐) does not affect the overall ratio of l‐ to d‐AHLs, that is d‐AHL production was not selectively enhanced by d‐methionine addition. However, the overall AHL (l‐ and d‐) concentration does increase with the addition of any methionine supplement. These findings indicate the possibility of a distinct biosynthetic pathway for d‐AHL production, possibly exposing a new dimension within bacterial communication.

## Graphical abstract

Production of d, l‐*N*‐acyl‐homoserine lactones (AHLs) by *Burkholderia cepacia* and *Vibrio fischeri* was monitored over time, with significant amounts of d‐AHLs detected. Quorum sensing (QS) of *V. fischeri* was observed with maximum bioluminescence correlating with the maximum concentrations of both l‐ and d‐ octanoyl‐homoserine lactones (l‐ and d‐OHL).

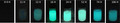

## 1 INTRODUCTION

Bacteria perceive and react to their surroundings by chemical signals (Taga & Bassler, 2003). When faced with environmental stress or changes, bacterial cells can act quickly, adapting, either by changing their structure, physiology, or behavior for survival. Two landmark papers, on bacterial signaling, proposed that bacteria communicate with each other via produced signaling molecules (Nealson et al., 1970; Tomasz, 1965) called autoinducers. This phenomenon, now known as quorum sensing (QS), has been extensively studied and is reasonably well understood (Fuqua et al., 1994; Mukherjee & Bassler, 2019; Whiteley et al., 2017). QS is described as the bacteria's ability to sense their population based on the concentration of autoinducers present in their environment. When a critical amount has been reached, the bacteria will jointly express a phenotype. Bioluminescence in *Vibrio fischeri* or biofilm production in *Burkholderia cepacia* are examples of two phenotypes that can be expressed (Lewenza et al., 1999; Li et al., 2018).

**FIGURE 1:**
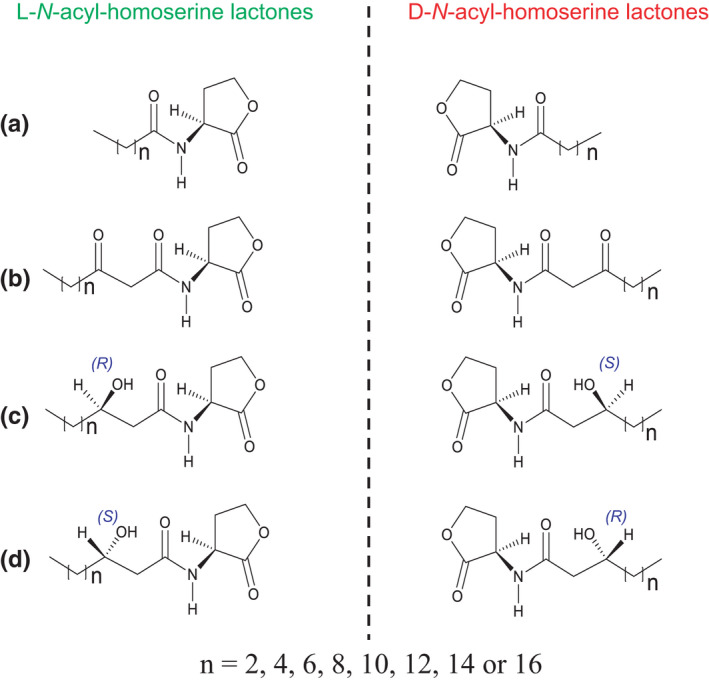
Structures of *N*‐acyl‐homoserine lactone (AHL) enantiomers. The structures are for (a) acyl, (b) 3‐oxoacyl, and (c) and (d) 3‐hydroxyacyl. The structures of (c) and (d) differ in stereochemistry on carbon 3 of the acyl chain

*N*‐acyl‐homoserine lactones (AHLs) are a widely studied class of autoinducer molecules in Gram‐negative bacteria (Fuqua & Greenberg, 2002). The biological precursor for AHLs is S‐adenosylmethionine (SAM). Figure 1 shows the general structure of AHLs which consists of a γ‐butyrolactone moiety with an acylated α‐amino group. The acyl chain length varies from 4 to 14 carbons. However, longer chain lengths up to 18 carbons have been reported (Mohamed et al., 2008). The α‐carbon on the lactone ring is a stereogenic center. Chemically, AHLs can exist in either the l‐ or d‐ configuration. It is well known that chiral proteogenic amino acids in all organisms are primarily l‐amino acids. Until recently, bacteria were believed to produce only the l‐form of AHLs for QS. This belief led researchers to conduct studies using, almost exclusively, achiral methods (Liu et al., 2018). Most AHL analyses from bacterial cultures are currently conducted using nonenantioselective methods, such as: thin‐layer chromatography combined with biosensor strains, GC‐MS, LC‐MS, or SFC‐MS (Charlton et al., 2000; Gao et al., 2020; Hoang et al., 2020; Zhou et al., 2020). To the best of our knowledge, only one study has mentioned the presence of d‐AHL, that is, d‐decanoyl‐homoserine lactone detected in a culture of *B*. *cepacia* LA3. The experiment used a single drop microextraction technique with chiral GC‐MS (Malik et al., 2009). They postulated that bacteria could biosynthesize d‐AHL as well as precursor d‐methionine derivatives. Other studies have shown the biosynthesis of d‐amino acids (Armstrong et al., 1993a; Armstrong et al., 1993b; Hernández & Cava, 2016; Weatherly et al., 2017).

A thorough investigation of the natural production of all manner of d‐AHLs and l‐AHLs is necessary to better understand the QS process. In this study, wild‐type *V*. *fischeri* (ES114) and *B*. *cepacia* (25416) were cultured, and the production of both enantiomers of AHLs was monitored over time using different nutrient conditions. *Vibrio fischeri* classically has been a point of reference in QS studies. It plays an active role in oceanic environments, having a symbiotic relationship with the bobtail squid (Koehler et al., 2019). Previously, the production of l‐AHLs from bacterial cultures was monitored over time and compared to bacterial growth (OD_600_) (Fekete et al., 2010; Ravn et al., 2001; Widdel, 2007). These studies did not apply chiral methods for the analysis of AHLs as it was incorrectly assumed that only l‐AHLs were produced. This study will present the first growth versus time studies that focus on the production of both l and d‐AHLs from *V*. *fischeri* and *B*. *cepacia*. Additionally, nutrient conditions are altered, for *B*. *cepacia*, by supplementation with l‐methionine, d‐methionine, or racemic methionine, to investigate whether or not different stereochemical forms of this amino acid affects the production of l and d‐AHLs.

## 2 MATERIALS AND METHODS

### 2.1 Analytical reagents

Racemic standards of *N*‐hexanoyl‐homoserine lactone (HHL), *N*‐heptanoyl‐homoserine lactone (HpHL), *N*‐octanoyl‐homoserine lactone (OHL), *N*‐decanoyl‐homoserine lactone (DHL), and *N*‐dodecanoyl‐homoserine lactone (dDHL), *N*‐(3‐oxohexanoyl)‐homoserine lactone (OHHL), M9 broth, d‐(+)‐glucose BioReagent grade, magnesium sulfate BioReagent grade, dl‐methionine, and all HPLC grade solvents were obtained from Sigma‐Aldrich, (St. Louis, Missouri, USA). *N*,*O*‐bis(trimethylsilyl)trifluoroacetamide (BSTFA) with 1% v/v trimethylchlorosilane (TMCS), 1 gram Supel^TM^‐select HLB SPE (HLB, hydrophilic‐lipophilic balance) cartridges, and β‐DEX^™^ 225 (25% 2,3‐di‐*O*‐acetyl‐6‐*O*‐TBDMS‐β‐cyclodextrin in SPB‐20 poly(20% phenyl/80% dimethylsiloxane) column, 30 m x 0.25 mm i.d. (film thickness 0.25 μm) were obtained from Millipore Sigma (Supelco, Bellefonte, Pennsylvania, USA). Photobacterium broth was obtained from Carolina Biological (Burlington, North Carolina, USA). Deionized water was obtained from an in‐house Millipore system producing water of 18.2 MΩ•cm resistivity.

### 2.2 Strains and growth conditions for bacteria

Two Gram‐negative bacteria that are known to produce AHLs were selected, *B*. *cepacia*, 25416, from ATCC (Manassas, VA) and *V*. *fischeri*, ES114 from Carolina Biological (Burlington, NC). Stock cultures of these strains were stored in their respective media with 25% glycerol at −80°C. Frozen *B*. *cepacia* strains were inoculated in M9 broth containing 0.4% d‐glucose and 2 mM magnesium sulfate (creating M9 media) and grown overnight (18 h) at 30°C (200 rpm). The overnight culture was then seeded to fresh M9 media grown at 30°C (200 rpm) for subsequent studies. Frozen *V*. *fischeri* strains were inoculated in photobacterium broth (PBB) media at 25°C (200 rpm). The overnight culture was then seeded to fresh PBB media grown at 25°C (200 rpm) for subsequent studies. The pH of all growth cultures from the beginning to the end of the experiments was always between 7.2–7.4.

### 2.3 Analysis of standard *N*‐acyl‐homoserine lactones by chiral gas chromatography‐mass spectrometry (GC‐MS)

A 100 μL aliquot of a 10 ppm standard solution of acyl and 3‐oxoacyl‐HLs in dichloromethane (C4‐C12 acyl; C4‐C8 3‐oxohexanoyl) was placed in a 1 mL sample vial and dried with a gentle stream of ultrahigh purity (UHP) N_2_. Derivatization of 3‐oxoacyl‐HLs was conducted as outlined in the literature (Readel et al., 2020). A 100 μL aliquot of BSTFA with 1% TMCS was added to the sample vial with the dried standard solution and sealed with a PTFE lined cap. The sample vial was placed into a 130°C preheated sand bath for 45 min. The sand bath was equilibrated at this temperature for 3 h prior to use in a GC oven. The sample was then removed from the oven and 100 μL of dichloromethane was added to the derivatization solution and analyzed on an Agilent 6890N GC System with 5975 MSD single quadrupole (Agilent Technologies, Palo Alto, CA, USA) equipped with a β‐DEX 225^TM^ column. The inlet split ratio was 100:1 (220°C), 1 μL injection, with an oven temperature program starting at 160°C (5‐min hold) increased at a rate of 1°C/min to 230°C (25‐min hold), constant flow of 1.1 mL/min (40 cm/s). The MS temperature conditions were as follows: MS transfer line at 230°C, ion source temperature of 280°C at 70 eV, and quadrupole temperature of 150°C. The MS was operated in selected ion monitoring (SIM) mode for ion 143 m/z (acyl‐homoserine lactones) and 185 m/z (for OHHLs).

### 2.4 Extraction reproducibility of *N*‐acyl‐homoserine lactones (AHL) standards from bacterial media

Fresh M9 media and PBB were both spiked with a 2 μg/mL solution of standard AHLs. Only PBB media was spiked with 3‐oxohexanoyl‐homoserine lactone (OHHL). The spiked media samples were then extracted using 1 g HLB cartridges which were sequentially conditioned with 5 mL of HPLC grade methanol and 5 mL of DI water. Ten mL of the spiked media sample was then loaded on the cartridge bed at a rate of ~2 mL/min. Loaded samples were then washed with a solution of 95:5 v/v% water:methanol at a rate of ~2 mL/min. Elution was done twice with 4 mL of HPLC grade acetonitrile. Eluted samples were dried by rotary evaporation on a BUCHI R‐100 Rotavapor unit (BUCHI, Flawil, Switzerland) attached to a vacuum in a heated water bath (50°C). M9 media samples were reconstituted in 100 μL of dichloromethane and analyzed by GC‐MS. PBB samples were processed and analyzed by the above‐mentioned method. The method limit of detection (LOD) for acyl was approximately 0.001 μg/mL while for 3‐oxoacyl‐HLs was 0.006 μg/mL. Good extraction reproducibility of AHLs was seen in M9 minimal (M9) media and photobacterium broth (PBB). The %RSD of recovery for the spiked AHLs standards were all lower than 3%. Only l and d‐OHHL showed %RSD of recovery at 6.4% in PBB.

### 2.5 Analysis of enantiomeric AHLs from bacterial cultures by chiral GC‐MS

*N*‐acyl‐homoserine lactones were extracted and analyzed from preliminary 24‐h cultures of *B*. *cepacia* and *V*. *fischeri* by using the above‐mentioned culturing, extraction, and GC‐MS analysis methods. The 3‐oxohexanoyl‐homoserine lactone (OHHL) and 3‐oxooctanoyl‐homoserine lactone (OOHL) concentrations were below the LOD for the GC‐MS in bacterial samples. Therefore, OHHL and OOHL in the samples were quantified by LC‐MS/MS as previously reported (Readel et al., 2020).

### 2.6 Quantitative analysis

*Burkholderia cepacia*: Internal standard calibration curves were constructed by preparing standard solutions of HHL, OHL and DHL at concentrations of 0.2, 0.4, 0.8, 2.0, 2.4, 3.6, and 4 μg/mL in triplicate in HPLC grade acetonitrile and spiked with l‐dDHL to a concentration of 8 μg/mL and then analyzed by GC‐MS.

*Vibrio fischeri*: internal standard calibration curves were constructed by preparing standard solutions of HHL, OHL, and OHHL at concentrations of 0.80, 0.30, 1.25, 2.0, 2.5, 3.0, 5.0, 8.0, 12.0, 15.0, 22.5, 30.0 μg/mL in triplicate in HPLC grade acetonitrile and spiked with l‐HpHL to a concentration of 3 μg/mL. Samples were blown with UHP N_2_ until dried. The dried residue was derivatized and treated as described previously.

### 2.7 Growth and AHL production by *Burkholderia cepacia*

Overnight cultures of *B*. *cepacia* and *V*. *fischeri* were seeded to fresh M9 media and PBB media respectively and grown using the above‐mentioned conditions. Quadruplicate samples were evaluated for *B*. *cepacia* and *V*. *fischeri*. *Burkholderia cepacia*: a 1.5 mL aliquot was taken from the growth cultures at times: 4.5, 15, 20, 30, 45, 54, 69, 93 h, diluted by half with fresh M9 media, and placed into polystyrene cuvettes. OD_600_ absorbance measurements were taken to monitor bacterial growth with a Vernier SpectroVis^™^ Plus spectrophotometer. Two 10 mL samples were taken from the growth culture and centrifuged, only supernatant was set aside. The supernatant of each sample was spiked with l‐dodecanoyl‐homoserine lactone (l‐dDHL) to a concentration of 8.0 μg/mL and guided through the SPE method described in Section 2.4. The extractant was rotary evaporated to dryness. The leftover residue was dissolved into 200 μL of dichloromethane and transferred to a 2 mL sample vial for analysis by GC‐MS.

### 2.8 Growth, AHL, and bioluminescence production by *Vibrio fischeri*

A 1.5 mL aliquot was taken from the growth cultures at times: 6, 9, 10, 11, 12, 16, 20, 24, 28, 33.5, 44.5, 52, 60, and 74 h, diluted by half with fresh PBB media and placed in polystyrene cuvettes. OD_600_ measurements were taken as described in the previous section. Replicate 10 mL samples were taken per growth culture and processed in the same way as *B*. *cepacia*. The supernatant of each sample was spiked with l‐heptanoyl‐homoserine lactone (l‐HpHL) to a concentration of 3 μg/mL and extracted using the SPE method. After rotary evaporation, the residue was dissolved in 200 μL of dichloromethane, transferred to a 2 mL sample vial, and dried by blowing with UHP N_2_. The residue was derivatized via the method described in Section 2.4 and analyzed by GC‐MS. The bioluminescence intensities of *V*. *fischeri* were analyzed by using Image J from the National Institute of Health, USA (http://imagej.nig.gov.ij). First, the vial image was selected, and a “Plot Profile” option was used to generate an intensity plot of gray values as a function of position on the bioluminescence image. The larger the “gray value,” the higher is the intensity of the bioluminescence. Further documentation and calculation of gray values are provided in the software. The highest gray values from the “Plot Profile” are listed in the Results, Section 3.2.

### 2.9 Methionine enantiomers as nutrients for *Burkholderia cepacia*

Overnight cultures of *B*. *cepacia* were grown as described previously and supplemented with one of three amounts of methionine: (i) 6.8 mmol of l‐methionine (ii) 6.8 mmol of d‐methionine and (iii) 3.4 mmol each of l and d‐methionine, grown at 30°C (200 rpm). Collection of OD_600_ and *N*‐acyl‐homoserine lactone growth curves was done following the method for *B*. *cepacia* in Section 2.7.

## 3 RESULTS

### 3.1 Presence of both enantiomeric forms of AHLs from bacterial cultures

**FIGURE 2:**
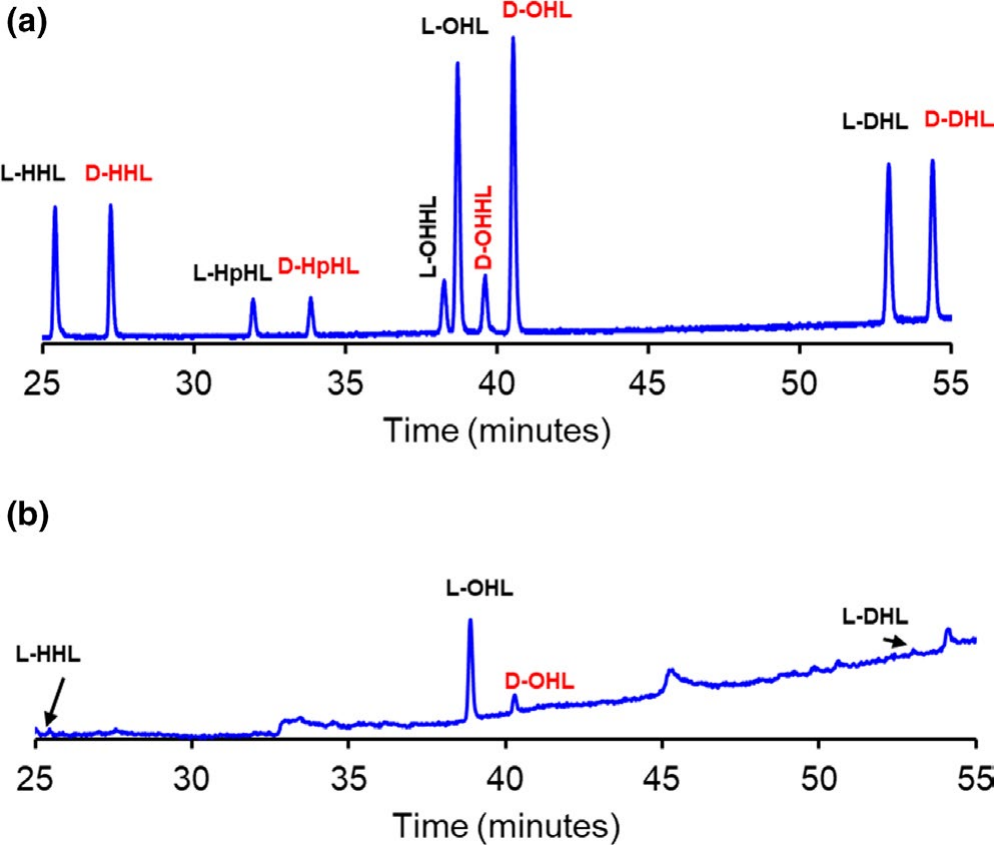
(a) GC‐MS chromatogram of a standard solution of D and L hexanoyl (HHL), heptanoyl (HpHL, internal standard), octanoyl (OHL), decanoyl (DHL), 3‐oxobutanoyl (OBHL), and **b**. GC‐MS chromatogram showing the production of l‐HHL, d‐ and l‐OHL, and possibly l‐DHL from a 24‐h growth culture of *Burkholderia cepacia*. (See experimental for GC‐MS and extraction methods)

**FIGURE 3:**
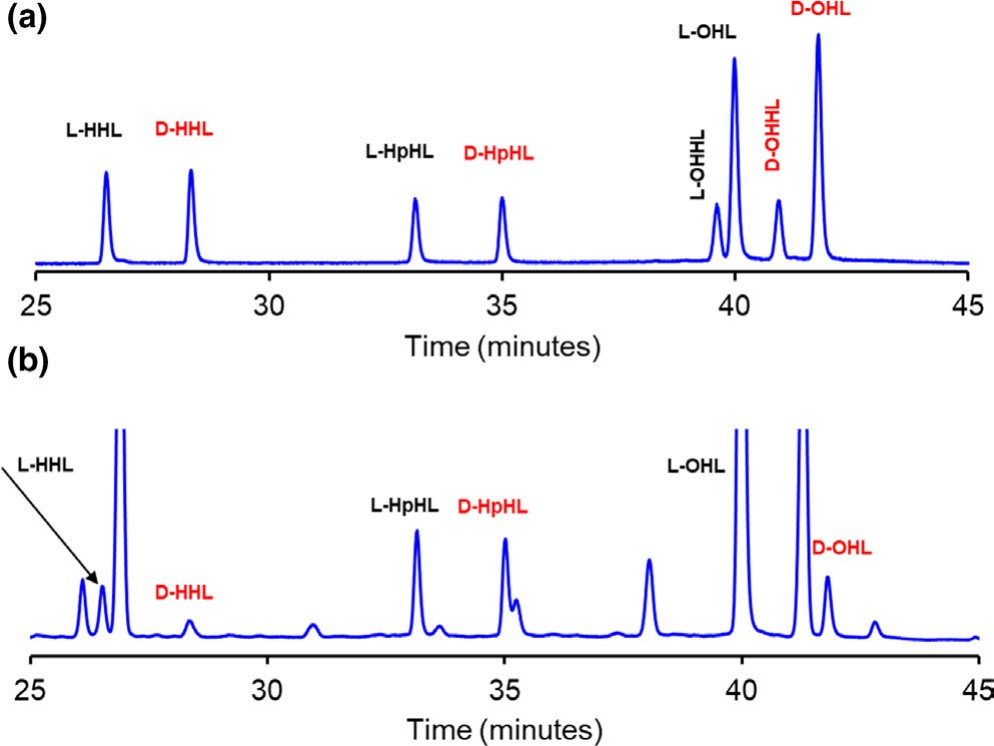
(a) GC‐MS chromatogram of a standard solution of dl‐hexanoyl (HHL), heptanoyl (HpHL), octanoyl (OHL), and 3‐oxohexanoyl homoserine lactones and (b) GC‐MS chromatogram showing the production of D and L isomers of HHL and OHL from 24‐h growth culture of *Vibrio fischeri*. 3.0 μg/mL of D and L HpHL was used as an internal standard. Unlabeled peaks are culture peaks from media. (See Material and Methods for GC‐MS and extraction details)

The AHL standards shown in Table 1 were selected because the occurrence of such l‐AHLs is well‐documented in cultures of *B*. *cepacia* and *V*. *fischeri* (Kuo et al., 1994; Lewenza et al., 1999; Venturi et al., 2004). The focal point of this study is the stereochemical production of these AHLs over time. An initial 24‐h culture of *B*. *cepacia* was tested for the presence and identification of any AHLs produced. The detected AHLs were l‐HHL, l‐ and d‐OHL, and l‐DHL (Figure 2a,b). The amounts of l‐HHL and l‐DHL were near the limit of detection (LOD, *Experimental Procedures*). In a 24‐h preliminary culture of *V*. *fischeri*, the extracts contained substantial amounts of l‐ and d‐HHL and l‐ and d‐OHL (Figure 3a,b). The 3‐oxo‐AHLs, OHHL and OOHL, were detected at much smaller amounts for *V*. *fischeri*, and only by LC‐MS/MS. Table 2 summarizes the screening results. The most abundant AHL detected for both bacteria was l‐OHL with the next most abundant being d‐OHL. The amounts of l‐HHL and l‐DHL produced by *B*. *cepacia* were approximately the same. However, *V*. *fischeri* produced approximately three times higher concentrations of l‐HHL compared to d‐HHL (Figure 3b). Other peaks in the extracted samples did not match with any of the standard AHLs used in this study.

**TABLE 1:** *N*‐acyl‐homoserine lactones (AHL) standards analyzed and the ion detected for analysis.

*N*‐acyl‐homoserine lactones^a^		
AHL	Ion detected (m/z, GC, LC)^b^	Bacterial Production (B, V)^c^
HHL	143,‐	B, V
HpHL	143,‐	B
OHL	143,‐	B, V
DHL	143,‐	B
dDHL	143,‐	‐
OHHL	185,102	V
OOHL	‐,102	‐

^a^ N‐hexanoyl‐homoserine lactone (HHL), N‐heptanoyl‐homoserine lactone (HpHL, internal standard) N‐octanoyl‐homoserine lactone (OHL), N‐decanoyl‐homoserine lactone (DHL), N‐dodecanoyl‐homoserine lactone (dDHL), N‐(3‐oxohexanoyl)‐homoserine lactone (OHHL), and N‐(3‐oxooctanoyl)‐homoserine lactone.

^b^ GC and LC denote the analytical method used to detect the respective AHLs (see Materials and Methods).

^c^ *B. cepacia* (B), *V. fischeri* (V). The dash (‐) denotes that the production of AHL is not documented for either of the bacteria studied.

**TABLE 2:** Chiral *N*‐acyl‐homoserine lactone (AHL) production patterns for *B*. *cepacia* grown in M9 media, three methionine‐supplemented *B*. *cepacia* also grown in M9 media, and *Vibrio fischeri* was grown in photobacterium broth. Dots (•) represent detectable homoserine lactones. (See experimental for details).

	Detected *N*‐acyl‐homoserine lactones^a^											
	l‐HHL	d‐HHL	l‐HpHL	d‐HpHL	l‐OHL	d‐OHL	l‐DHL	d‐DHL	l‐OHHL	d‐OHHL	l‐OOHL	d‐OOHL
*Burkholderia cepacia*	•				•	•	•					
*B. cepacia (l‐met)*	•	•			•	•	•					
*B. cepacia (d‐met)*	•	•			•	•	•					
*B. cepacia (d,l‐met)*	•	•			•	•	•					
*Vibrio fischeri*	•	•			•	•			•^b^		•^b^	

^a^ *N*‐hexanoyl‐homoserine lactone (HHL), *N*‐heptanoyl‐homoserine lactone (HpHL, internal standard) *N*‐octanoyl‐homoserine lactone (OHL), *N*‐decanoyl‐homoserine lactone (DHL), *N*‐(3‐oxohexanoyl)‐homoserine lactone (OHHL), and *N*‐(3‐oxooctanoyl)‐homoserine lactone.

^b^ These were detected only by LC‐MS/MS.

### 3.2 Chiral *N*‐acyl‐homoserine lactone (AHL) production during bacterial growth

**FIGURE 4:**
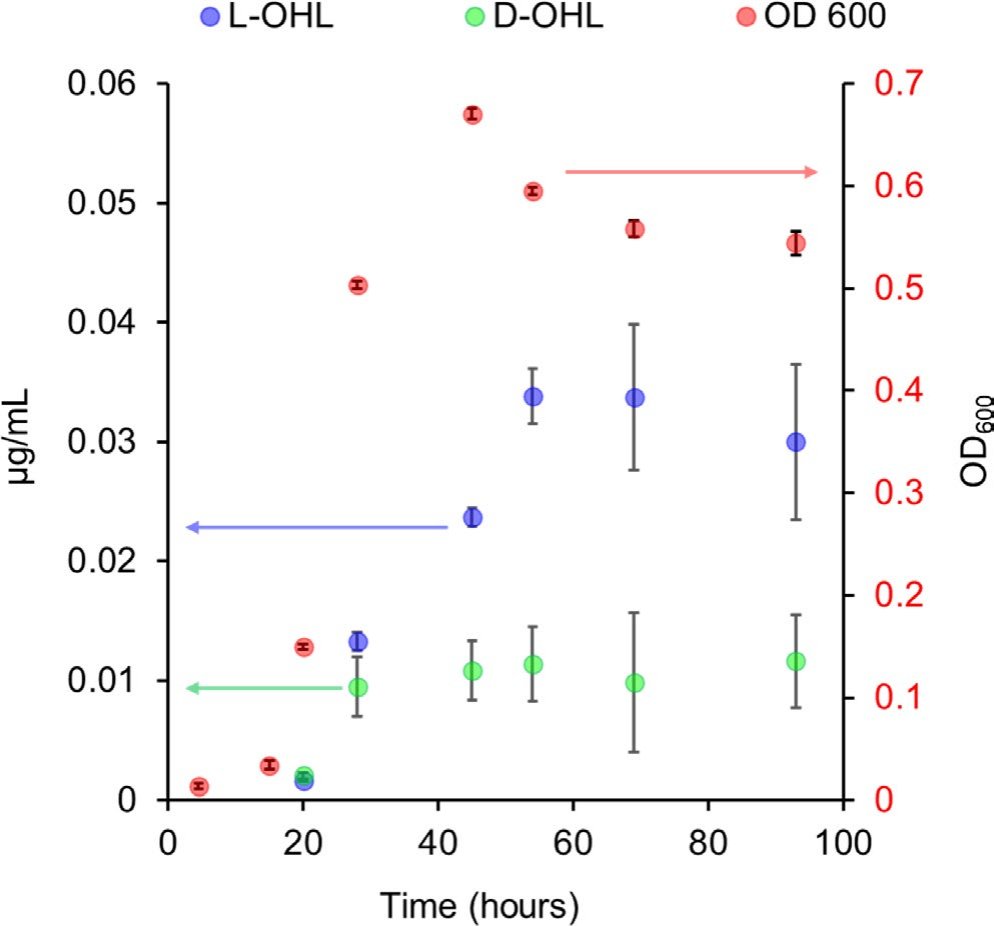
Production of d,l‐octanoyl‐homoserine lactone (OHL) with biomass (OD_600_) curves during growth of *B*. *cepacia*. Data represent averages with standard deviations calculated from four replicates

Figure 4 shows the OD_600_ growth curve for *B*. *cepacia*. An increase in cell density is observed over time with a maximum reached at ~45 h. Cell density decreased slightly at 54 h and then remained nearly constant through the end of the experiment. Initial production of the dominant l‐OHL was observed at 20 h. The l‐OHL production increased until reaching a maximum concentration of 0.034 μg/mL at ~54 h. The enantiomer, d‐OHL, was initially detected at ~20 h, which was the same time of appearance as l‐OHL. The concentration of d‐OHL reached a maximum at ~30 h, with a concentration of approximately 0.01 μg/mL, which remained at that level for the duration of the experiment (Figure 4). Other AHLs detected were l‐HHL and l‐DHL. Both were initially detected at 30 h, with a maximum concentration of approximately 0.007 μg/mL for both at ~70 h.

**FIGURE 5:**
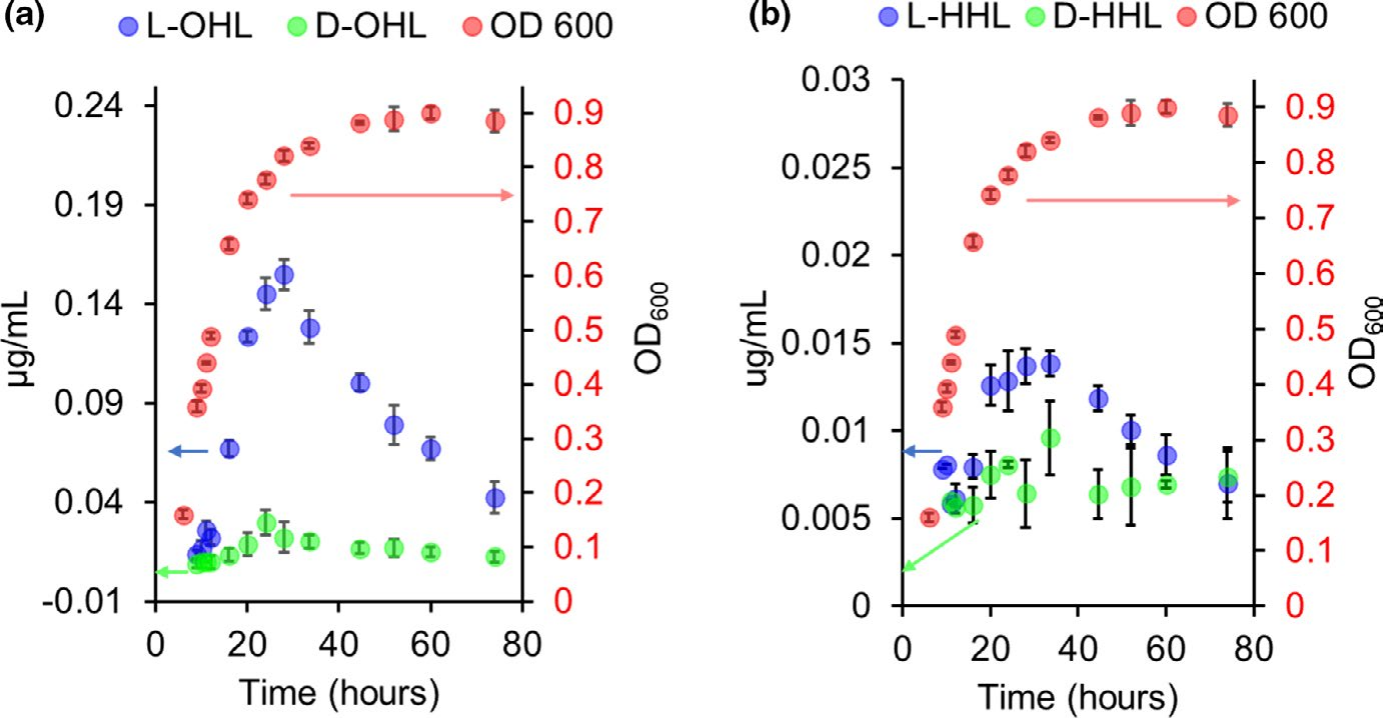
Production of (a) d,l‐octanoyl‐homoserine lactone (OHL) and (b) d,l‐hexanoyl‐homoserine lactone (HHL) with biomass (OD_600_) curves during growth of *V*. *fischeri*. Data represent averages with standard deviations calculated from four replicates

The OD_600_ growth curve for *V*. *fischeri* also showed increasing cell density with a maximum cell density observed at ~44 h. The observed cell density then remained constant through the end of the experiment (Figure 5a,b). l‐OHL concentrations reached a maximum of 0.150 μg/mL at ~28 h. Significant depletion of l‐OHL was observed at ~33 h, with its concentration diminishing to 0.043 μg/ml by the end of the experiment (Figure 5a). d‐OHL was first detected at ~9 h and a maximum concentration of 0.030 μg/mL was reached at ~24 h. There were also signs of slight depletion of d‐OHL starting at ~33 h after which the concentration plateaued at 0.010 μg/mL. Both l and d‐HHL were observed for *V*. *fischeri* with greater amounts observed for the l‐enantiomer (Figure 5b). The concentration l‐HHL steadily increased over time, reaching a level of 0.014 μg/mL at ~33 h. The d‐HHL also was produced at slightly lower concentrations, reaching a maximum of 0.009 μg/mL at ~33 h. Much lower amounts of l‐3‐oxo‐HHL (OHHL) and l‐3‐oxo‐OHL (OOHL) were detected by LC‐MS/MS (Readel et al., 2020) at concentrations of approximately 0.0001 μg/mL for both enantiomers.

**FIGURE 6:**
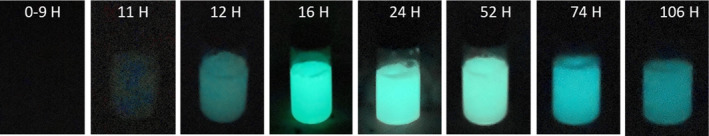
Images of bioluminescence of *V*. *fischeri* over time, cultured in photobacterium broth (PBB) at 25°C. Relative intensities at the indicated time intervals are: 0 h = 0, 11 h = 24, 12 h = 88, 16 h = 167, 24 h = 193, 52 h = 190, 74 h = 153, 106 h = 100. See Materials and Methods section for experimental details

Bioluminescence was monitored throughout the growth of *V*. *fischeri* (Figure 6). The initial observation of bioluminescence by *V*. *fischeri* was at 11 h with maximum luminescence (light production) seen at approximately 24 h. A gradual dimming (decreasing light production) was observed starting at 28 h and continued to the end of the experiment (Figure 6). The maximum luminescence correlated with the maximum production of d‐OHL and l‐OHL at ~24–28 h (Figure 5a). It is important to note that the most produced AHL was l‐OHL and the second most produced was its enantiomer d‐OHL. All other l‐AHLs were of lower concentrations than d‐OHL for both *B*. *cepacia* and *V*. *fischeri*.

### 3.3 Enantiomeric *N* *‐acyl‐homoserine lactone production by* *Burkholderia cepacia* in methionine‐supplemented M9 media

**FIGURE 7:**
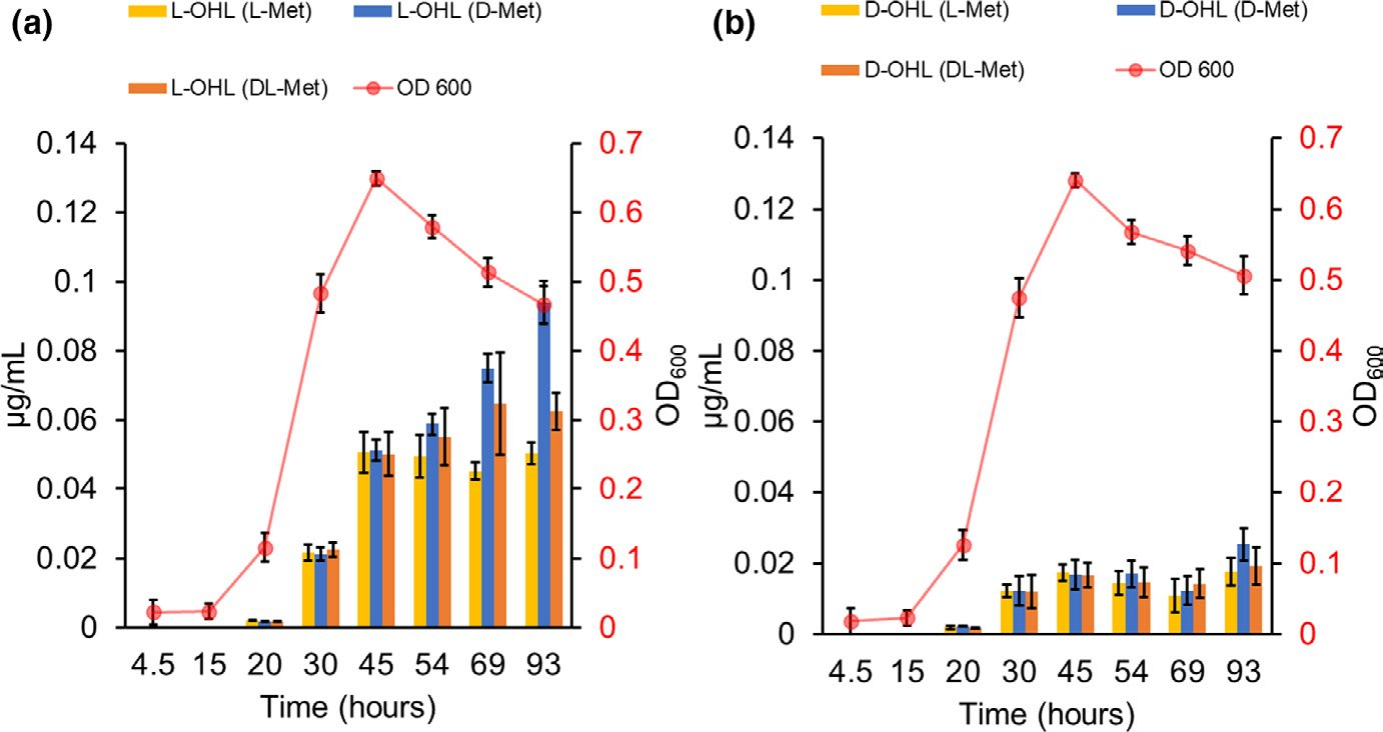
Production of (a) l‐octanoyl‐homoserine lactone (l‐OHL) and (b) d‐octanoyl‐homoserine lactone (d‐OHL) with biomass (Abs OD 600) curves during growth of *B*. *cepacia* supplemented with 6.8 mmol of l‐methionine (l‐Met), 6.8 mmol of d‐methionine (d‐Met) or supplemented with 3.4 mmol of l‐methionine and 3.4 mmol of d‐methionine (d,l‐Met). Data represent averages with standard deviations calculated from four replicates

There has only been one instance where a d‐AHL has been reported from a bacterial culture. d‐decanoyl‐homoserine lactone was detected in a culture of *B*. *cepacia*. (Malik et al., 2009) The authors postulated that the production of this molecule “... could be derived from its biosynthesis since it forms from amino acid derivatives and the bacteria were shown to be able to produce d‐methionine derivatives.” (Malik et al., 2009). To see if the presence of methionine enantiomers influenced AHL production, parallel, identical, cultures of *B*. *cepacia* were individually supplemented with identical amounts of l‐Met, d‐Met, or racemic Met (d,l‐Met). The production of l‐ and d‐OHL was compared to the unsupplemented culture of Figure 4. The three supplemented cultures had comparable growth curves, reaching maximum cell densities at ~45 h (Figure 7a,b). Subsequently a slight, but continual, decrease in cell density was found until the end of the experiment (Figure 7). Again, it was observed that l‐OHL was the dominant AHL produced for all methionine‐supplemented cultures of *B*. *cepacia* (Figure 7a). The l‐Met supplemented cultures produced 0.051 μg/mL of l‐OHL at 45 h with its concentration staying constant through the end of the growth period. Interestingly, the production of l‐OHL for the d‐Met supplemented cultures increased continuously to the end of the experiment reaching a concentration of 0.094 μg/mL at ~93 h (Figure 7a). For d,l‐Met supplemented cultures, production of l‐OHL reached a maximum concentration of 0.065 μg/mL at ~70 h (Figure 7a). The maximum production of d‐OHL was approximately 0.020 μg/mL for all three of the parallel cultures (Figure 7b). Production of l‐HHL and l‐DHL was observed at lower concentrations than the aforementioned AHLs. Their concentrations reached a maximum of approximately 0.008 μg/mL for both AHLs. The production of d‐HHL was observed for all parallel, methionine supplemented, cultures, reaching maximum concentrations of approximately 0.006 μg/mL. d‐HHL was not previously observed (for unsupplemented *B*. *cepacia* cultures) as it was below the detection limit of the method. Comparing the results to those of the unsupplemented culture it is clear that the addition of any form of methionine (l‐, d‐, or racemic) does not affect the ratio of l‐ to d‐AHL production, that is to say, d‐AHL production is not exclusively enhanced by any addition of methionine. However, methionine supplementation does increase the overall AHL concentrations (both l‐ and d‐AHLs).

## 4 DISCUSSION

*Burkholderia cepacia* (25416) and *V*. *fischeri* (ES114) were found to produce d‐AHLs throughout their growth period. All d‐AHLs detected were enantiomers of the expected l‐AHLs that are produced by these two species (Fuqua et al., 1994; Kuo et al., 1994; Lewenza et al., 1999; Venturi et al., 2004). It is known that the AHL responsible for the regulation of the *lux* operon and bioluminescence in *V*. *fischeri* is OHHL (Fuqua et al., 1994; Kuo et al., 1994). However, the amounts of OHL produced were ~1500 times higher than OHHL for this strain of *V*. *fischeri* during this study. It has been observed that ES114 produces OHL at higher amounts than OHHL (Stabb et al., 2008). Nevertheless, bioluminescence of *V*. *fischeri* correlated with the most produced AHLs, l‐ and d‐OHL. The role of l‐OHL in *V*. *fischeri* and its involvement in bioluminescence has been explored by others. These studies conclude that the role of l‐OHL is as an initial inducer of bioluminescence and regulator of OHHL production in the *lux* system of *V*. *fischeri* (Collins et al., 2005; Lupp & Ruby, 2004; Miyashiro & Ruby, 2012; Sun et al., 2018).

d‐OHL was the second most produced AHL both by *V*. *fischeri* and by *B*. *cepacia* cultured in M9 media (Figure 4). All other AHLs were produced in much lower amounts. Although pH‐mediated chemical hydrolysis could decrease the total AHLs, this would not affect the relative amounts of AHL enantiomers that have identical chemical hydrolysis rates. Further, chemical hydrolysis at the pH encountered in these studies was minimal (pH 7.2 to 7.4) (see Materials and Methods, Section 2.2). Questions arise as to both the mechanism of production and the possible biological significance of the ubiquitous d‐AHLs. One suggested biosynthetic pathway was that d‐AHLs are produced via the same process as the l‐AHLs, but perhaps with a d‐methionine derivative starting material. Another possibility is that, initially, the AHLs produced are of the l‐configuration and they are subsequently acted upon by a racemase enzyme. Amino acid racemases, including ones for serine and methionine, are well‐known throughout many biological systems (Radkov & Moe, 2014), but no AHL racemases have been reported, to our knowledge.

An examination of parallel cultures of *B*. *cepacia* supplemented with methionine (l‐, d‐, or racemic) produced much higher concentrations of AHLs, l‐ and d‐ alike, with the detection of d‐HHL now possible. The d‐Met supplemented cultures produced the highest concentrations of AHLs, about three times more than the unsupplemented media. d,l‐Met, and l‐Met produced about two times and 1.5 times the amounts of AHLs as compared to the unsupplemented cultures (Figure 7 vs Figure 4). The ratios of the l‐AHLs to their corresponding d‐AHL enantiomer did not significantly change regardless of the stereochemical nature of the methionine supplement. It is known that most bacterial species can biosynthesize methionine, meaning it is not an essential amino acid (Ferla & Patrick, 2014). It appears that any added methionine (ie, l‐, d‐ or d,l‐) to the *B*. *cepacia* cultures was mainly used as an alternative carbon and nitrogen source in the M9 minimal media. The fact that l‐Met supplemented cultures produced lower amounts of all AHLs than d‐Met cultures was surprising. Perhaps l‐Met has immediate utility in the organism's other metabolic pathways (Tanaka et al., 1985), while d‐Met is more broadly useful as a carbon and nitrogen source.

d‐AHL production was not exclusively enhanced by the addition of any of the methionine stereoisomers. In the biosynthesis of l‐AHLs, l‐methionine is used to make S‐adenosylmethionine, with this metabolite transforming into various l‐AHLs when acylated by an acyl‐carrier protein. It appears that d‐AHLs may not be directly produced by the conventional biosynthetic pathway of l‐AHLs. d‐methionine does not seem to play a direct role in the production of d‐AHLs. Auto‐racemization studies have been applied to AHLs, with findings suggesting there is negligible physico‐chemical racemization occurring in the bacterial culture nor during the chromatographic time frame (Hodgkinson et al., 2011; Malik et al., 2009). There are at least two other possibilities for the origin of d‐AHLs, another unidentified synthetic pathway, distinct from that of l‐AHL synthesis, or the aforementioned possibility of chiral interconversion between l‐ and d‐AHLs.

Collins et al. (2005) studied the directed evolution of *V*. *fischeri* LuxR as a means to broaden the spectrum and sensitivity to different l‐AHLs. They found that broadening a given protein's specificity is the easiest evolutionary path to obtaining higher selectivity to new signaling molecules. However, their focus was on mutations that affected or were affected by the nature of the acyl group, but not on the stereochemistry of the homoserine lactone group. This is not to say that an analogous mechanism may not be relevant for d‐AHLs, only that it hasn't been considered or studied. d‐AHLs are known to have acted as autoinducer molecules (Li et al., 2018). However, it also is possible that they play some other, as yet undetermined, regulatory or feedback role in quorum sensing.

## 5 CONCLUSIONS

This work is the first to detect d‐AHLs in the wild‐type strain of *V*. *fischeri* (ES114) and the first to monitor the production of d‐AHLs over time for *B*. *cepacia* (25416) and *V*. *fischeri* (ES114). The AHL screening and production curves demonstrated that d‐OHL and d‐HHL are produced in significant amounts by both bacteria. In *V*. *fischeri*, maximum bioluminescence correlated with maximum concentrations of both d‐OHL and l‐OHL. The addition of any enantiomer of methionine, into M9 minimal media, augments the production of all AHLs (l‐ and d‐) by *B*. *cepacia*. However, the d‐ to l‐ ratios of AHLs are not affected. The supplementation of the *B*. *cepacia* growth media with d‐Met enhanced the production of both l and d‐AHLs to a greater extent than the addition of equivalent amounts of l or d,l‐Met. The study indicates that another biosynthetic pathway is likely involved in d‐AHL production. Alternatively, the initially produced l‐AHL is converted to the d‐AHL. The reasons for the ubiquitous presence of d‐AHLs are considered but are not yet well understood.

## CONFLICT OF INTEREST

None declared.

## AUTHOR CONTRIBUTIONS

**Abiud E. Portillo:** Conceptualization (supporting); Formal analysis (equal); Investigation (equal); Methodology (equal); Writing‐original draft (lead); Writing‐review & editing (equal). **Elizabeth Readel:** Formal analysis (supporting); Investigation (supporting); Methodology (equal); Writing‐review & editing (supporting). **Daniel W. Armstrong:** Conceptualization (lead); Formal analysis (equal); Funding acquisition (lead); Methodology (supporting); Project administration (lead); Supervision (lead); Writing‐review & editing (supporting).

## ETHICS STATEMENT

None required.

## Data Availability

Data generated and analyzed during this study are provided in this publication.

## References

[mbo31242-bib-0001] Armstrong, D. W. , Gasper, M. P. , Lee, S. H. , Ercal, N. , & Zukowski, J. (1993). Factors controlling the level and accurate determination of D‐amino acids in the urine and plasma of laboratory rodents. Amino Acids, 5, 299–315. 24190673 10.1007/BF00805992

[mbo31242-bib-0002] Armstrong, D. W. , Gasper, M. P. , Lee, S. H. , Zukowski, J. , & Ercal, N. (1993). D‐amino acid levels in human physiological fluids. Chirality, 5, 375–378. 10.1002/chir.530050519. 8398594

[mbo31242-bib-0003] Charlton, T. S. , De Nys, R. , Netting, A. , Kumar, N. , Hentzer, M. , Givskov, M. , & Kjelleberg, S. (2000). A novel and sensitive method for the quantification of N‐3‐oxoacyl homoserine lactones using gas chromatography–mass spectrometry: Application to a model bacterial biofilm. Environmental Microbiology, 2(5), 530–541. 11233161 10.1046/j.1462-2920.2000.00136.x

[mbo31242-bib-0004] Collins, C. H. , Arnold, F. H. , & Leadbetter, J. R. (2005). Directed evolution of *Vibrio fischeri* LuxR for increased sensitivity to a broad spectrum of acyl‐homoserine lactones. Molecular Microbiology, 55(3), 712–723. 10.1111/j.1365-2958.2004.04437.x. 15660998

[mbo31242-bib-0005] Fekete, A. , Kuttler, C. , Rothballer, M. , Hense, B. A. , Fischer, D. , Buddrus‐Schiemann, K. , & Hartmann, A. (2010). Dynamic regulation of *N‐*acyl‐homoserine lactone production and degradation in Pseudomonas putida IsoF. FEMS Microbiology Ecology, 72(1), 22–34. 20100181 10.1111/j.1574-6941.2009.00828.x

[mbo31242-bib-0006] Ferla, M. P. , & Patrick, W. M. (2014). Bacterial methionine biosynthesis. Microbiology, 160(8), 1571–1584. 10.1099/mic.0.077826-0. 24939187

[mbo31242-bib-0007] Fuqua, C. , & Greenberg, E. P. (2002). Listening in on bacteria: acyl‐homoserine lactone signalling. Nature Reviews Molecular Cell Biology, 3(9), 685–695. 10.1038/nrm907. 12209128

[mbo31242-bib-0008] Fuqua, W. C. , Winans, S. C. , & Greenberg, E. P. (1994). Quorum sensing in bacteria: The LuxR‐LuxI family of cell density‐responsive transcriptional regulators. Journal of Bacteriology, 176(2), 269–275. 10.1128/jb.176.2.269-275.1994. 8288518 PMC205046

[mbo31242-bib-0009] Gao, X. Y. , Fu, C. A. , Hao, L. , Gu, X. F. , Wang, R. , Lin, J. Q. , & Lin, J. Q. (2020). The substrate‐dependent regulatory effects of the AfeI/R system in Acidithiobacillus ferrooxidans reveals the novel regulation strategy of quorum sensing in acidophiles. Environmental Microbiology, 23(2), 757–773. 32656931 10.1111/1462-2920.15163 PMC7984328

[mbo31242-bib-0010] Hernández, S. B. , & Cava, F. (2016). Environmental roles of microbial amino acid racemases. Environmental Microbiology, 18(6), 1673–1685. 10.1111/1462-2920.13072. 26419727

[mbo31242-bib-0011] Hoang, T. P. T. , Barthélemy, M. , Lami, R. , Stien, D. , Eparvier, V. , & Touboul, D. (2020). Annotation and quantification of *N‐*acyl homoserine lactones implied in bacterial quorum sensing by supercritical‐fluid chromatography coupled with high‐resolution mass spectrometry. Analytical and Bioanalytical Chemistry, 412, 1–16. 10.1007/s00216-019-02265-4. 31919609

[mbo31242-bib-0012] Hodgkinson, J. T. , Galloway, W. R. , Casoli, M. , Keane, H. , Su, X. , Salmond, G. P. , & Spring, D. R. (2011). Robust routes for the synthesis of *N‐*acylated‐L‐homoserine lactone (AHL) quorum sensing molecules with high levels of enantiomeric purity. Tetrahedron Letters, 52(26), 3291–3294. 10.1016/j.tetlet.2011.04.059.

[mbo31242-bib-0013] Koehler, S. , Gaedeke, R. , Thompson, C. , Bongrand, C. , Visick, K. L. , Ruby, E. , & McFall‐Ngai, M. (2019). The model squid–vibrio symbiosis provides a window into the impact of strain‐and species‐level differences during the initial stages of symbiont engagement. Environmental Microbiology, 21(9), 3269–3283. 10.1111/1462-2920.14392. PMC6386636 30136358

[mbo31242-bib-0014] Kuo, A. , Blough, N. V. , & Dunlap, P. V. (1994). Multiple *N‐*acyl‐L‐homoserine lactone autoinducers of luminescence in the marine symbiotic bacterium *Vibrio fischeri* . Journal of Bacteriology, 176(24), 7558–7565. 10.1128/jb.176.24.7558-7565.1994. 8002580 PMC197213

[mbo31242-bib-0015] Lewenza, S. , Conway, B. , Greenberg, E. , & Sokol, P. A. (1999). Quorum sensing in *Burkholderia cepacia*: identification of the LuxRI homologs CepRI. Journal of Bacteriology, 181(3), 748–756. 9922236 10.1128/jb.181.3.748-756.1999 PMC93439

[mbo31242-bib-0016] Li, S.‐Z. , Xu, R. , Ahmar, M. , Goux‐Henry, C. , Queneau, Y. , & Soulère, L. (2018). Influence of the d/l configuration of *N‐*acyl‐homoserine lactones (AHLs) and analogues on their Lux‐R dependent quorum sensing activity. Bioorganic Chemistry, 77, 215–222. 10.1016/j.bioorg.2018.01.005. 29367078

[mbo31242-bib-0017] Liu, J. , Fu, K. , Wu, C. , Qin, K. , Li, F. , & Zhou, L. (2018). “In‐Group” communication in marine vibrio: A review of *N‐*acyl homoserine lactones‐driven quorum sensing. Frontiers in Cellular and Infection Microbiology, 8, 139. 10.3389/fcimb.2018.00139. 29868495 PMC5952220

[mbo31242-bib-0018] Lupp, C. , & Ruby, E. G. (2004). *Vibrio fischeri* LuxS and AinS: Comparative study of two signal synthases. Journal of Bacteriology, 186(12), 3873–3881. 15175301 10.1128/JB.186.12.3873-3881.2004 PMC419941

[mbo31242-bib-0019] Malik, A. K. , Fekete, A. , Gabifuegi, I. , Rothballer, M. , & Schmitt‐Kopplin, P. (2009). Single drop microextraction of homoserine lactones based quorum sensing signal molecules, and the separation of their enantiomers using gas chromatography mass spectrometry in the presence of biological matrices. Microchimica Acta, 166(1), 101–107. 10.1007/s00604-009-0183-x.

[mbo31242-bib-0020] Miyashiro, T. , & Ruby, E. G. (2012). Shedding light on bioluminescence regulation in *Vibrio fischeri* . Molecular Microbiology, 84(5), 795–806. 10.1111/j.1365-2958.2012.08065.x. 22500943 PMC3359415

[mbo31242-bib-0021] Mohamed, N. M. , Cicirelli, E. M. , Kan, J. , Chen, F. , Fuqua, C. , & Hill, R. T. (2008). Diversity and quorum‐sensing signal production of Proteobacteria associated with marine sponges. Environmental Microbiology, 10(1), 75–86. 18211268 10.1111/j.1462-2920.2007.01431.x

[mbo31242-bib-0022] Mukherjee, S. , & Bassler, B. L. (2019). Bacterial quorum sensing in complex and dynamically changing environments. Nature Reviews Microbiology, 17(6), 371–382. 10.1038/s41579-019-0186-5. 30944413 PMC6615036

[mbo31242-bib-0023] Nealson, K. H. , Platt, T. , & Hastings, J. W. (1970). Cellular control of the synthesis and activity of the bacterial luminescent system. Journal of Bacteriology, 104(1), 313–322. 10.1128/jb.104.1.313-322.1970. 5473898 PMC248216

[mbo31242-bib-0024] Radkov, A. D. , & Moe, L. A. (2014). Bacterial synthesis of D‐amino acids. Applied Microbiology and Biotechnology, 98, 5363–5374. 10.1007/s00253-014-5726-3. 24752840

[mbo31242-bib-0025] Ravn, L. , Christensen, A. B. , Molin, S. , Givskov, M. , & Gram, L. (2001). Methods for detecting acylated homoserine lactones produced by Gram‐negative bacteria and their application in studies of AHL‐production kinetics. Journal of Microbiological Methods, 44(3), 239–251. 10.1016/S0167-7012(01)00217-2. 11240047

[mbo31242-bib-0026] Readel, E. , Portillo, A. , Talebi, M. , & Armstrong, D. W. (2020). Enantiomeric separation of quorum sensing autoinducer homoserine lactones using GC‐MS and LC‐MS. Analytical and Bioanalytical Chemistry, 412, 2927–2937. 10.1007/s00216-020-02534-7. 32193589

[mbo31242-bib-0027] Stabb, E. , Schaefer, A. , Bose, J. , & Ruby, E. (2008). Quorum signaling and symbiosis in the marine luminous bacterium *Vibrio fischeri* . In S. C. Winans , B. L. Bassler , C. C. A. Bacteria , & D. C. Washingon (Eds.), Chemical Communication Among Bacteria, Washingon, DC, ASM Press 233–250.

[mbo31242-bib-0028] Sun, H. , Pan, Y. , Gu, Y. , & Lin, Z. (2018). Mechanistic explanation of time‐dependent cross‐phenomenon based on quorum sensing: A case study of the mixture of sulfonamide and quorum sensing inhibitor to bioluminescence of Aliivibrio fischeri. Science of the Total Environment, 630, 11–19. 10.1016/j.scitotenv.2018.02.153. 29471187

[mbo31242-bib-0029] Taga, M. E. , & Bassler, B. L. (2003). Chemical communication among bacteria. Proceedings of the National Academy of Sciences, 100(suppl 2), 14549–14554. 10.1073/pnas.1934514100. PMC304117 12949263

[mbo31242-bib-0030] Tanaka, H. , Esaki, N. , & Soda, K. (1985). A versatile bacterial enzyme: L‐methionine γ‐lyase. Enzyme and Microbial Technology, 7(11), 530–537. 10.1016/0141-0229(85)90094-8.

[mbo31242-bib-0031] Tomasz, A. (1965). Control of the competent state in Pneumococcus by a hormone‐like cell product: an example for a new type of regulatory mechanism in bacteria. Nature, 208(5006), 155–159. 5884251 10.1038/208155a0

[mbo31242-bib-0032] Venturi, V. , Friscina, A. , Bertani, I. , Devescovi, G. , & Aguilar, C. (2004). Quorum sensing in the *Burkholderia cepacia* complex. Research in Microbiology, 155(4), 238–244. 10.1016/j.resmic.2004.01.006. 15142620

[mbo31242-bib-0033] Weatherly, C. A. , Du, S. , Parpia, C. , Santos, P. T. , Hartman, A. L. , & Armstrong, D. W. (2017). D‐Amino acid levels in perfused mouse brain tissue and blood: A comparative study. ACS Chemical Neuroscience, 8, 1251–1261. 28206740 10.1021/acschemneuro.6b00398 PMC5479723

[mbo31242-bib-0034] Whiteley, M. , Diggle, S. P. , & Greenberg, E. P. (2017). Progress in and promise of bacterial quorum sensing research. Nature, 551(7680), 313–320. 29144467 10.1038/nature24624 PMC5870893

[mbo31242-bib-0035] Widdel, F. (2007). Theory and measurement of bacterial growth. Didalam Grundpraktikum Mikrobiologie, 4(11), 1–11.

[mbo31242-bib-0036] Zhou, J. , Lin, Z. J. , Cai, Z. H. , Zeng, Y. H. , Zhu, J. M. , & Du, X. P. (2020). Opportunistic bacteria use quorum sensing to disturb coral symbiotic communities and mediate the occurrence of coral bleaching. Environmental Microbiology, 22(5), 1944–1962. 10.1111/1462-2920.15009. 32249540

